# NeuO-mediated O-acetylation of uropathogenic *Escherichia coli* K1 capsule enhances resistance to phage and neutrophil killing

**DOI:** 10.1128/jb.00610-25

**Published:** 2026-02-10

**Authors:** Lachlan L. Walker, Nguyen Thi Khanh Nhu, Zheng Jie Lian, Kate M. Peters, Mercedes Monteleone, James P. R. Connolly, Mark J. Walker, Brian M. Forde, Kate Schroder, Matthew J. Sweet, Minh-Duy Phan, Mark A. Schembri

**Affiliations:** 1 Institute for Molecular Bioscience, The University of Queensland, Brisbane, Queensland, Australia; 2 Australian Infectious Diseases Research Centre, The University of Queensland 1974, Brisbane, Queensland, Australia; 3 Newcastle University Biosciences Institute, Newcastle University 105562, Newcastle upon Tyne, United Kingdom; 4 UQ Centre for Clinical Research, Faculty of Health and Behavioural Sciences, The University of Queensland 1974, Brisbane, Queensland, Australia; 5 School of Chemistry and Molecular Biosciences, The University of Queensland 1974, Brisbane, Queensland, Australia; Dartmouth College Geisel School of Medicine, Hanover, New Hampshire, USA

**Keywords:** uropathogenic *Escherichia coli*, capsule, phase variation, urinary tract infection, bacterial pathogenesis, bacterial virulence

## Abstract

Uropathogenic *Escherichia coli* (UPEC) strains that express the K1 capsule are associated with severe invasive disease, including pyelonephritis, urosepsis, and neonatal meningitis. The K1 capsule can be modified by NeuO, a phage-encoded phase-variable O-acetyltransferase. The role of O-acetylation in pathogenesis of K1 UPEC remains to be fully elucidated. Here, we assessed the prevalence of the *neuO* gene in a K1 *E. coli* data set comprising 8,659 genomes and observed that 43.5% of genomes harbor *neuO*, with a high prevalence (88.1%) in the pandemic UPEC clone sequence type (ST) 95. We generated an isogenic ∆*neuO* mutant in the reference ST95 strain MS7163 and complemented this with a phase-ON locked version of the *neuO* gene, resulting in constitutive O-acetylation of the K1 capsule. The phase-variable rate of *neuO* in MS7163 was measured by fragment analysis at 93% phase-ON with 21 heptanucleotide repeats. NeuO acetylated the K1 sialic acid into O-acetylated forms, including Neu5,7Ac2, Neu5,8Ac2, and Neu5,9Ac2. We demonstrate that O-acetylation increased survival of K1 UPEC to lytic K1 phage and human neutrophils, yet increased susceptibility to human serum. O-acetylation of the K1 capsule did not influence bladder colonization in a murine model of urinary tract infection. Overall, we hypothesize that phase-variable O-acetylation is a niche-specific adaptive mechanism that enhances survival of UPEC in different ways, including protection against K1 phage and resistance to neutrophil-mediated killing.

**IMPORTANCE:**

The K1 polysialic acid capsule is a key virulence factor of uropathogenic *Escherichia coli* (UPEC). A subset of K1 UPEC possesses the phage-encoded phase-variable *neuO* gene, which mediates O-acetylation of the capsule. However, the prevalence, phase dynamics, and biological consequences of this modification remain to be fully elucidated. Here, we show that the *neuO* gene exhibits variable distribution among K1 UPEC, with a high prevalence in the global ST95 clone and evidence for active phase switching. We further demonstrate that K1 O-acetylation confers resistance to phage and neutrophil killing, suggesting a role associated with enhanced survival in infection and environmental settings.

## INTRODUCTION

Urinary tract infections (UTIs) are one of the most common bacterial infections, with an estimated 400 million cases annually across the globe (1). Approximately 75% of all UTIs are caused by uropathogenic *Escherichia coli* (UPEC) (2). At the severe end of the UTI disease spectrum, acute pyelonephritis can be life-threatening and progress to sepsis. The polysaccharide capsule is an important UPEC virulence factor, and despite its extensive variation, some capsule types are more frequently associated with severe disease. The K1 capsule is particularly significant; longitudinal surveillance studies show that K1-encapsulated *E. coli* account for ~25% of all bloodstream infections and are associated with invasive diseases, including pyelonephritis and neonatal meningitis (3–6). UPEC strains that express the K1 capsule survive efficiently in human serum and blood (7, 8).

The K1 capsule is an α2,8-linked polysialic acid homopolymer that consists of 5-N-acetylneuraminic acid (Neu5Ac) building blocks (9). Polysialic acid of the K1 capsule can be recognized by host immune receptors, including Siglecs, and O-acetylation of the K1 capsule has been reported to modulate immune recognition (10, 11). O-acetylation is mediated by the phase-variable O-acetyltransferase gene *neuO*, a cargo gene of the CUS-3 bacteriophage (12–14). Phase variation of *neuO* is caused by DNA strand slippage of a heptanucleotide tandem repeat (5′-AAGACTC-3′) in the 5′ region of the coding sequence (13). Full-length translation of the protein-coding sequence occurs when the number of repeats is divisible by 3 (13).

The role of *neuO* in the pathogenesis of K1 *E. coli* remains to be fully elucidated. Some reports suggest that isolates with an O-acetylated K1 capsule have increased virulence and enhanced immune evasion in both *in vitro* and *in vivo* models (11, 15). In contrast, other studies have shown that O-acetylation does not provide a selective advantage in blood during *in vivo* animal studies and does not significantly affect adherence to human brain endothelial cells (16–18). Despite these conflicting reports, population-based studies show that 91% of human invasive *E. coli* K1 encapsulated strains and 43% of K1 encapsulated UTI strains harbor the *neuO* gene (19). Notably, 98% of sequence type complex 95 strains harbor *neuO* (19). Sequence type (ST) 95 is one of several major UPEC pandemic clones, including ST131, ST73, and ST69 (20). Given the broad conservation of *neuO* within the ST95 lineage, we aimed to explore if O-acetylation of the K1 capsule contributes to the success of this globally disseminated clone.

In this study, we analyzed a large collection of 8,659 publicly available *E. coli* genomes that contained the K1 capsule locus, which revealed 43.5% harbor the *neuO* gene. Molecular analysis demonstrated NeuO-mediated O-acetylation of the K1 capsule provides protection against lytic K1 phage. In terms of UPEC virulence, NeuO-mediated O-acetylation resulted in variable phenotypes; O-acetylation provided protection against killing by human neutrophils, yet increased susceptibility to human serum and did not alter bladder colonization in an experimental mouse model. This suggests that phase variation of *neuO* provides niche-specific advantages for K1 *E. coli*.

## MATERIALS AND METHODS

### Bioinformatic analyses

The data set consisting of 8,659 K1 *E. coli* genomes was identified from 192,234 complete and draft genomes (downloaded 13 December 2022 from the NCBI, Data S1), using Kaptive 3.0 (21) and the K1 capsule locus from the *E. coli* group 2 and 3 capsule database (22). Kaptive results that were considered “typeable” were reported (Data S1). Sequence types were determined by MLST (23), and genomes with an undefined ST were removed (24). Blastn was used to query this database for the genes of interest (25). To determine the prevalence of *neuO*, the *neuO* gene sequence from the MS7163 genome was used (accession number CP026853.1), with the variable 5′ heptanucleotide tandem repeat region excluded (26). The BLASTn results were filtered with the thresholds of >90% identity and >80% alignment length. Phylogroup labeling for STs was performed with data available on Enterobase (27).

### Bacterial strains and growth conditions

Bacteria were grown at 37°C on solid or in liquid lysogeny broth (LB) medium. Media were supplemented with chloramphenicol (30 μg/mL), kanamycin (50 μg/mL), or gentamicin (20 μg/mL) when appropriate. Bacterial strains used in this study are described in Table S1.

### Molecular methods

DNA extraction, purification, and Sanger sequencing were performed as previously described (28, 29). Primers used in this study are listed in Table S2. Fragment analysis was performed by the Genetic Research Services at the University of Queensland. Briefly, a primer was labeled with a 5′ 6-carboxyfluorescein (6-FAM) fluorophore. The subsequent PCR reaction labeled the PCR products with a 5′ 6-FAM fluorophore, and these samples were loaded onto the 3730 DNA analyzer (Thermo Fisher Scientific), which allowed for gel capillary electrophoresis to differentiate the sizes of the PCR product.

### Generation of isogenic mutant strains

The λ-Red recombinase system was used to generate a *neuO* mutant and to undertake subsequent chromosome complementation in MS7163 (30). To mutate the *neuO* gene, we performed a three-way PCR that involved fusing ~500 bp PCR products with overhangs from the upstream and downstream of the *neuO* gene to the chloramphenicol resistance gene cassette isolated from the pKD3 vector following a previously described method (31). The complement plasmid, pSU2718-pNeuO (pNeuO), carrying a *neuO* gene with a codon optimized 5′ region replacing the tandem repeats to prevent DNA slippage, resulting in a non-phase-variable (i.e., always ON) construct and a total of 39 heptanucleotide tandem repeats, was synthesized by Epoch Life Science, Inc. (Texas, USA). To perform chromosome complementation, a construct that contained the non-phase-variable version of *neuO* fused to the chloramphenicol cassette from the pKD3 plasmid was used. The non-phase-variable version of *neuO* and the chloramphenicol cassette were flanked by ~500 bp of upstream and downstream regions of the *neuO* gene using PCR as described (31). The resulting complementation construct was electroporated into MS7163∆*neuO*, followed by removal of the chloramphenicol cassette via the FLP recombinase system (30), resulting in MS7163*neuO^CC^*.

### Mass spectrometry of K1 capsule O-acetylation

K1 capsular sialic acid was extracted from overnight cultures and prepared using Signal DMB Labeling Kit (Agilent) (11). Time of flight mass spectrometry was performed on a Shimadzu Nexera uHPLC (Japan) at The University of Queensland Institute for Molecular Bioscience Mass Spectrometry facility.

### K1 phage killing assay

The K1 lytic phage vB_EcoM_SHAK7163 was used (32). Overnight bacterial cultures were standardized to an OD_600_ = 1 (~10^9^ colony-forming units [CFU]/mL) and infected with phage (2 × 10^8^ PFU/mL) at a multiplicity of infection (MOI) of 1:10 (phage:bacteria). Growth of bacteria was measured at 37°C with shaking at 200 rpm using a FLUOstar OPTIMA microplate reader (BMG Labtech).

### Neutrophil killing assay

Blood from a single donor was collected across multiple days using Vacuette tubes containing EDTA (Greiner Bio-One). Neutrophils were isolated using the EasySep Direct Human Neutrophil Isolation kit (EasySep). Purified neutrophils (~2.5 × 10^5^) were mixed with bacteria (2.5 × 10^6^ CFU) at an MOI of 1:10 at 37°C with shaking at 50 rpm and enumerated to determine CFU prior to incubation with neutrophils (time 0) and at 1 h post-incubation.

### Serum killing assay

Fresh blood from a single donor was collected using BD Vacutainer SST II Advanced tubes (BD). Isolated human serum (90 µL) was inoculated with bacteria (10 µL) at ~10^7^ CFU and incubated at 37°C for 1 h with shaking at 50 rpm. Bacteria were enumerated to determine CFU prior to incubation with human serum (time 0) and at 1 hour post-incubation.

### Mouse UTI model

All experiments were performed using the following housing conditions: (light:dark cycle 12:12 h, room temperature 21 ± 1°C, humidity 50 ± 10%). The mouse model of UTI was employed as previously described (33, 34). Briefly, female C57BL/6 mice (8–10 weeks) were infected via transurethral administration at 5 × 10^8^ CFU. Mice were euthanized by cervical dislocation at 24 h post-infection. Bacterial bladder loads were enumerated by plating onto LB agar. Mixed competitive infections were performed using a 1:1 mixture of strains tagged with different antibiotic resistance genes (chloramphenicol or kanamycin). Groups consisted of either wild-type MS7163 and MS7163∆*neuO*, or MS7163*neuO*^CC^ and MS7163∆*neuO*. Statistical analyses were performed using the two-tailed Wilcoxon matched-pairs signed-rank test (Prism 9, GraphPad).

## RESULTS

### The *neuO* gene is highly prevalent in the UPEC ST95 clone

**Fig 1:**
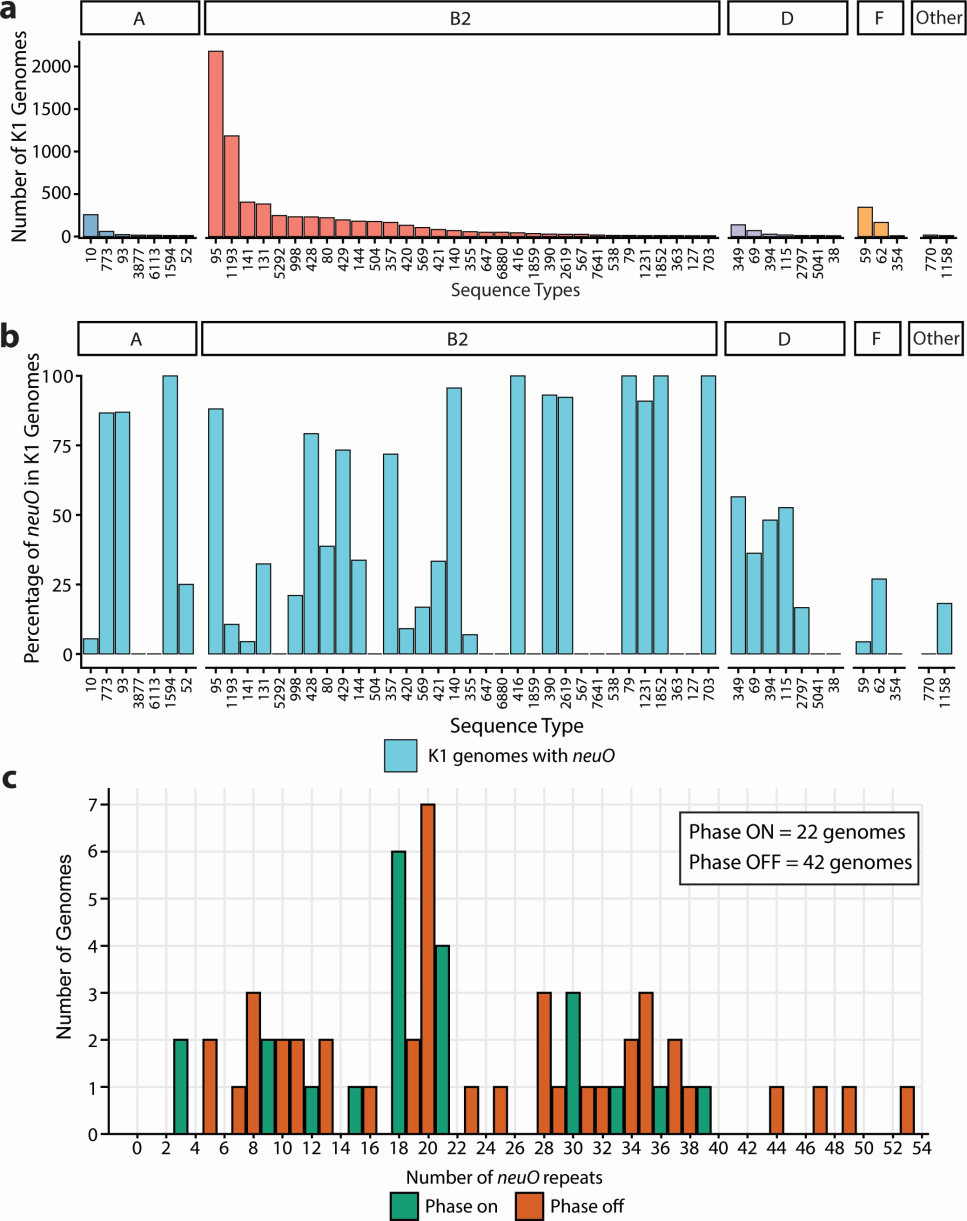
Prevalence of the *neuO* gene in 8,659 K1 *E. coli* genomes. (**a**) STs that contain at least ten K1 genomes are presented as a column with the y-axis indicating the total number of K1 genomes in each ST. The STs are split by phylogroups, with the phylogroups indicated above the column graph. (**b**) The percentage of genomes harboring *neuO* as determined by BLASTn (>90% identity and >80% alignment length thresholds) is shown in cyan. (**c**) The distribution of *neuO* repeats in complete genomes that harbored *neuO* (*n* = 64). Each column represents the number of repeats identified in each individual genome. The green color represents genomes with the number of repeats divisible by 3, while orange represents genomes not divisible by 3.

We curated a data set of 8,659 K1 genomes using Kaptive 3.0 (21) and screened for the presence of *neuO*. This analysis identified that the *neuO* gene is harbored in 43.5% of K1 genomes. To understand the distribution of *neuO* in K1 *E. coli*, we determined the STs of all K1 genomes and assessed the carriage of *neuO*. In total, 255 STs contained at least 1 K1-positive genome, and 51 STs contained 10 or more K1 genomes (Fig. 1a). The majority of STs with more than 10 K1 genomes belonged to phylogroups B2 and D (39/51) that primarily comprise UPEC and other extraintestinal pathogenic *E. coli* pathotypes (35). The STs with the highest number of K1-positive genomes were ST95 (2,181 genomes) and ST1193 (1,184 genomes); these STs accounted for 25.2% and 13.7% of total K1 genomes in our data set, respectively (Fig. 1a). The carriage of *neuO* among K1 STs with 10 or more genomes exhibited a variable distribution, ranging from 0% to 100% (Fig. 1b). The *neuO* gene was present at 85% or more in 12 STs (ST416, ST79, ST1594, ST1852, ST703, ST140, ST390, ST2619, ST1231, ST95, ST93, and ST773) (Fig. 1b). Within this group, ST95 was dominant, with 1,693/1,922 (88.1%) genomes positive for *neuO*. The remaining 11 STs comprised 10–66 K1 genomes. In contrast, *neuO* gene carriage varied among other large K1 lineages, including ST1193 (10.6%), ST141 (4.4%), ST131 (32.4%), and ST59 (4.3%). The *neuO* gene was absent in seven K1-positive STs (Fig. 1b). Overall, this analysis demonstrates that the prevalence of *neuO* is highly variable among K1 STs and primarily found in *E. coli* genomes from phylogroups B2 and D.

### Analysis of *E. coli* K1 complete genomes reveals a phase-variable genotype

To assess the phase status of *neuO*, we determined the proportion of K1 *E. coli* strains in the NCBI refseq complete genome database with phase “ON” versus “OFF” *neuO* genotypes. This data set comprised 64 completely sequenced K1 genomes that harbored *neuO*. These genomes were probed to identify the number of *neuO* heptanucleotide tandem repeats, revealing a range from 3 to 53 repeats, with 20 repeats being the most common and found in seven genomes (Fig. 1c). Of the 64 genomes that harbored *neuO*, 65.6% (42 genomes) were not in a multiple of three based upon the repeat region, indicative of a phase-OFF genotype (Fig. 1c).

### O-acetylation of the K1 capsule by NeuO confirmed by mass spectrometry

To dissect the role of *neuO* in O-acetylation of the K1 capsule, we utilized the ST95 UPEC strain MS7163. MS7163, which was isolated from a patient with acute pyelonephritis, belongs to the highly virulent neonatal meningitis-associated clone, O45:K1:H7 (5, 26). Long-read sequencing of MS7163 showed *neuO* was primarily in the phase-ON state with 21 heptanucleotide repeats, which was also verified by Sanger sequencing of the repeat region. To determine the phase status of MS7163 cells, we performed fragment analysis on four colonies, which revealed that 93% of the MS7163 cells contained 21 repeats, while the remainder contained 20 (1.92%) and 22 (5.35%) repeats, respectively. Thus, the majority of the MS7163 population is *neuO* phase-ON during laboratory culture in LB broth.

**Fig 2:**
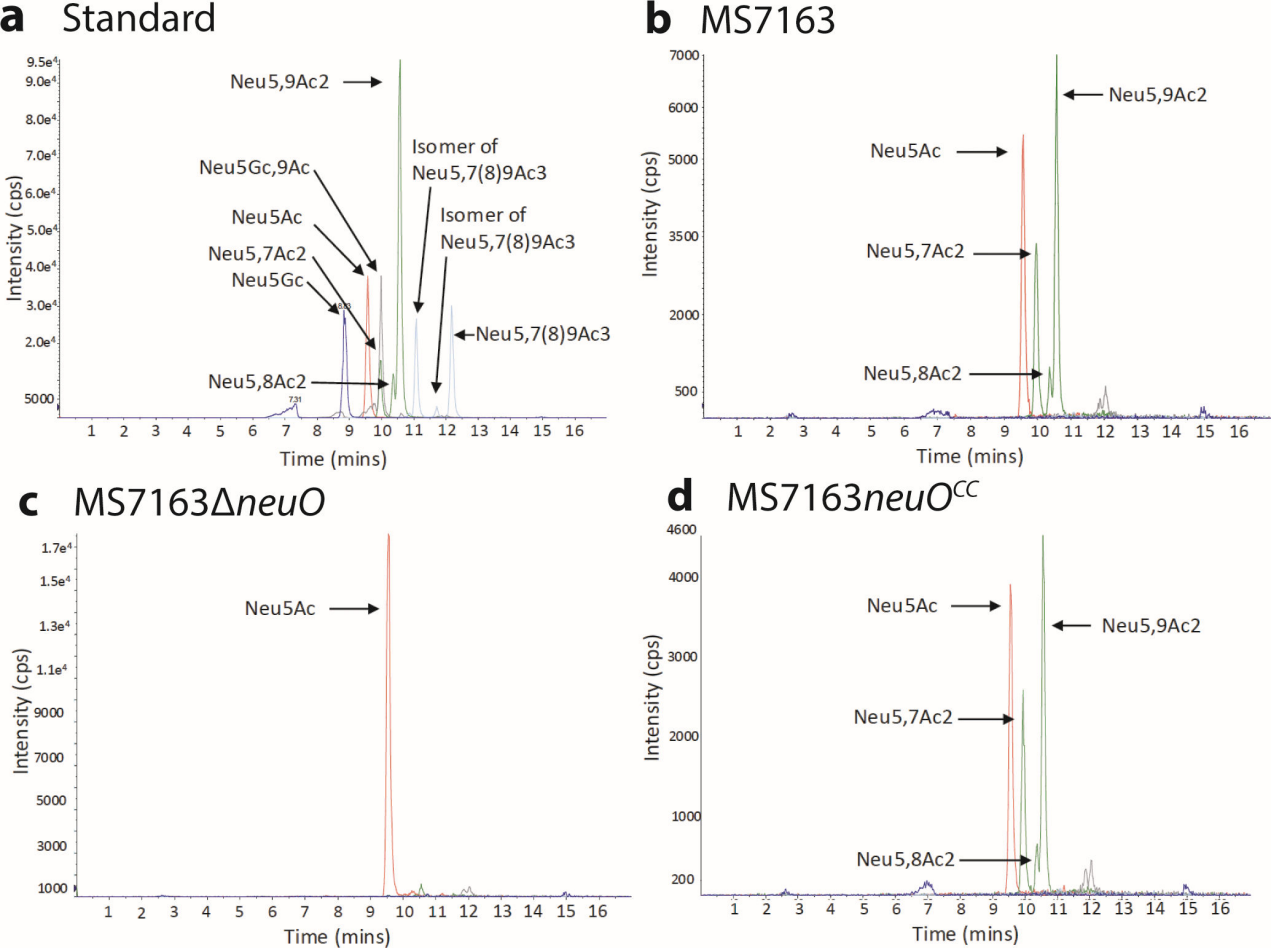
Mass spectrometry of K1 capsule O-acetylation. (**a**) Standards for the DMB sialic acid labeling kit (Agilent) with the corresponding peaks labeled. The subsequent panels represent mass spectrometry data for (**b**) wild-type MS7163, (**c**) MS7163∆*neuO*, and (**d**) MS7163*neuO*^CC^.

Next, we mutated the *neuO* gene in MS7163 using λ-Red recombination to generate the strain MS7163∆*neuO*. We also generated a chromosomally complemented *neuO* phase-ON locked strain (referred to as MS7163*neuO^CC^*). This strain was constructed by codon-optimizing the 5′ repeat region of the *neuO* gene to abolish the tandem repeat pattern (thereby preventing DNA strand slippage) and increase the size of the repeat region to 39 heptanucleotide tandem repeats (in line with previous literature indicating the length of the *neuO* repeat region increases the catalytic activity of NeuO [36]). To confirm the O-acetylation status of the K1 capsule in these isogenic strains, we used mass spectrometry with known O-acetylated sialic acid standards (Fig. 2a). The capsule of wild-type MS7163 exhibited peaks for Neu5Ac (sialic acid) as well as the O-acetylated forms of sialic acid: Neu5,7Ac2, Neu5,8Ac2, and Neu5,9Ac2 (Fig. 2b). MS7163∆*neuO* did not exhibit O-acetylation of the capsule, demonstrated by the single peak for Neu5Ac (Fig. 2c), while the MS7163*neuO^CC^* strain possessed an O-acetylation pattern consistent with successful complementation (Fig. 2d).

### NeuO is important for protection against lytic K1 phage

**Fig 3:**
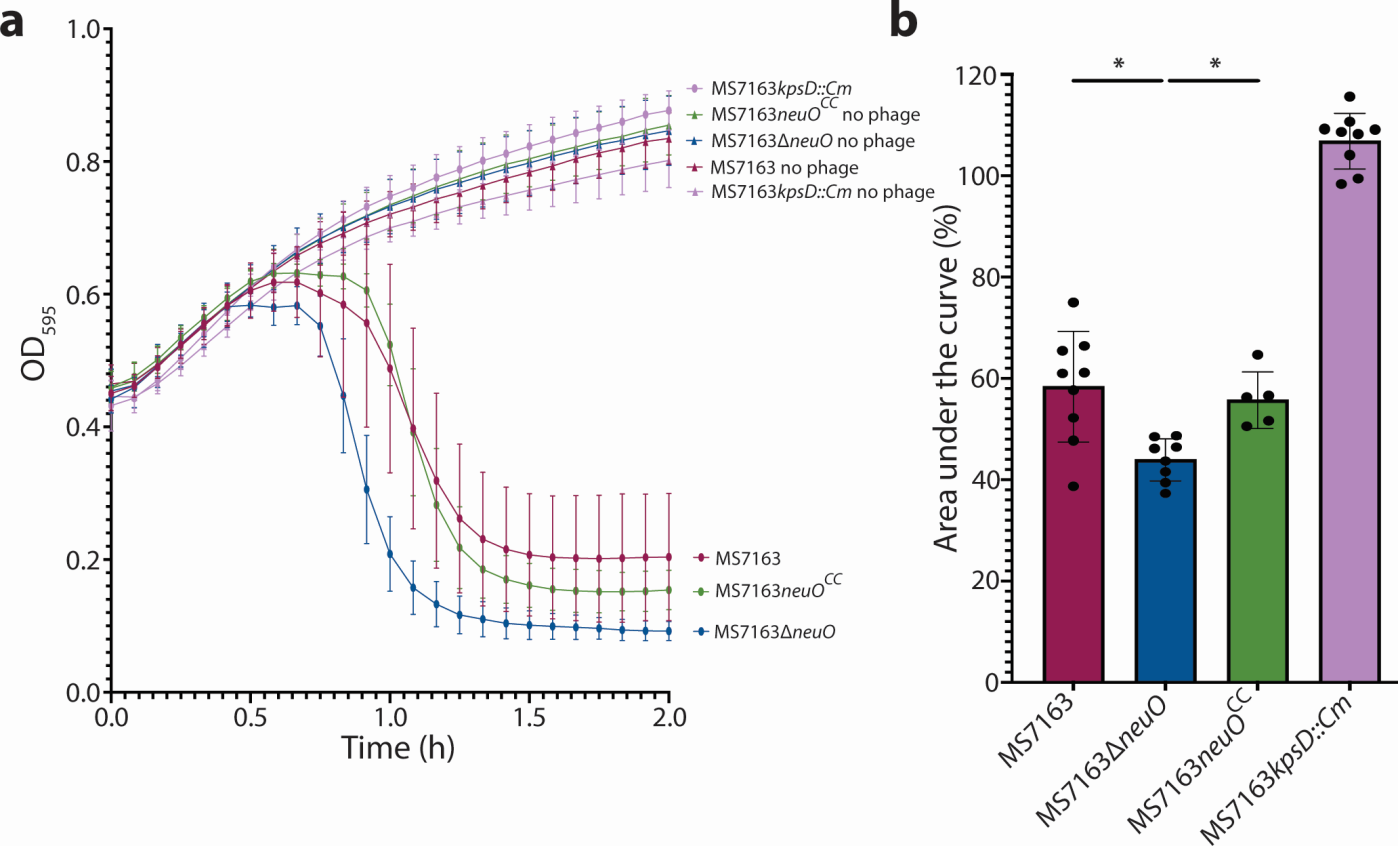
K1-specific lytic phage killing assay. (**a**) Growth of bacteria over 2 h in LB media with or without vB_EcoM_SHAK7163 phage (no phage control). Data represent the mean ± SD. Data for wild-type MS7163 and MS7163*kpsD* represent nine biological replicates, while data for MS7163∆*neuO* and *MS7163neuO^CC^* represent eight and five biological replicates, respectively. (**b**) Area under the curve analysis is presented as a bar graph, representing mean ± SD. GraphPad Prism was used to visualize data and determine statistical significance by Brown-Forsythe and Welch one-way ANOVA. Significance level is indicated by an asterisk: *P ≤ 0.05.

To investigate the effect of O-acetylation on K1 capsule recognition, we utilized the K1-specific lytic phage vB_EcoM_SHAK7163 (32). Strains MS7163, MS7163∆*neuO*, and MS7163*neuO*^CC^ were grown to mid-exponential phase, at which time phage was added to the culture and the optical density was tracked over 2 h. In these experiments, MS7163*∆neuO* was more susceptible to phage lysis compared to the O-acetylated strains MS7163 and MS7163*neuO^CC^* (Fig. 3). As a control, an MS7163 strain mutated for capsule production (MS7163*kpsD::Cm*) was also incubated with phage in the same manner; this mutant was not susceptible to phage lysis, demonstrating the K1 capsule specificity of the phage (Fig. 3). Together, these results support a previous analysis that suggested O-acetylation of the K1 capsule protects K1 *E. coli* from K1 phage recognition and lysis (16).

### O-acetylation does not enhance K1 UPEC bladder colonization

**Fig 4:**
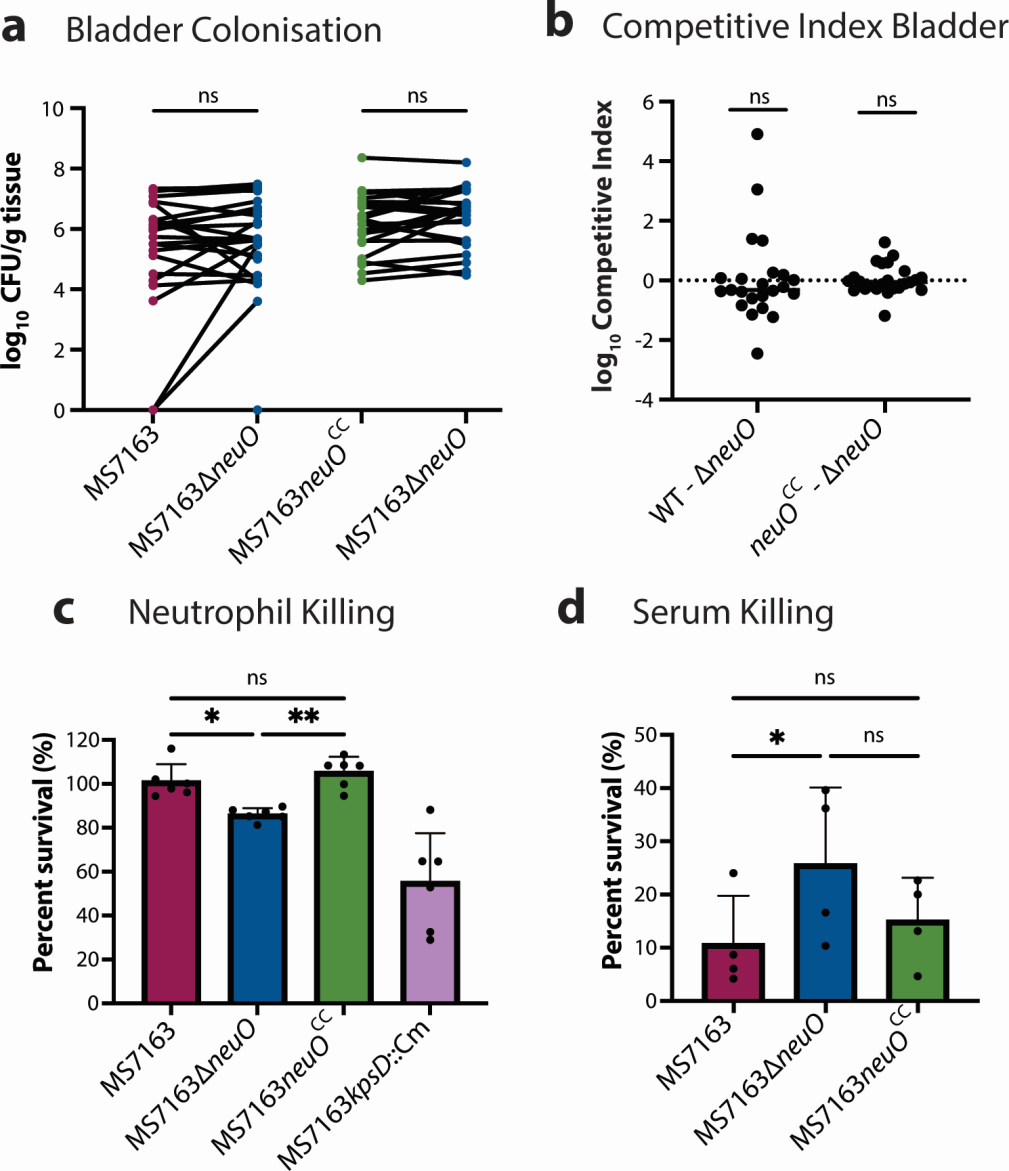
Infection impacts of K1 capsule O-acetylation. The mixed 50:50 infection consisted of two groups of strains (*n* = 24 mice per group). The first group contained MS7163 (WT) and MS7163∆*neuO* (∆*neuO*), and the second group contained MS7163*neuO*^CC^ (*neuO^CC^*) and MS7163∆*neuO*. (**a**) Data from the bladder are represented as CFU count. (**b**) Competitive index that indicates the ratio of CFU between strains in the mixed infection before and after infection. GraphPad Prism was used to visualize the data. To determine statistical significance, the Wilcoxon matched-pair signed-rank test was used for CFU, and the Wilcoxon signed-rank test with a hypothetical mean of 0 was used for CI. (**c**) Human neutrophil killing assay, with survival of bacteria after 1-h incubation with human neutrophils being plotted as a bar graph. Data represent mean ± SD from six biological replicates, and statistical significance was determined using Brown-Forsythe and Welch ANOVA. (**d**) Serum killing assay, showing mean percentage survival of bacteria ± SD after 1-h incubation with human serum. Data represent matched data of *n* = 4 biological replicates. Statistical significance was determined by Friedman’s test with Dunn’s multiple comparisons to account for day-to-day variation in serum activity. Data were visualized in GraphPad Prism. Significance levels are indicated by asterisks: *P ≤ 0.05, **P ≤ 0.01; ns = *P* > 0.05.

MS7163 has been shown to effectively colonize the bladder of experimentally infected mice (26, 28). To determine if O-acetylation of the K1 capsule impacts UPEC bladder colonization, C57BL/6 mice were transurethrally infected with a 1:1 mixed ratio of an O-acetylation-positive and O-acetylation-negative strain tagged with different antibiotic resistance markers to enable differential selection and comparative enumeration of bacterial loads. Two sets of mixed infections were performed. Groups consisted of wild-type MS7163 and MS7163∆*neuO*, or MS7163*neuO^CC^* and MS7163*∆neuO*. In these experiments, no significant difference in colonization of the bladder was observed for either group (Fig. 4a). This was also reflected by analysis of the competitive index for both groups, which showed no significant difference at 24 h post-infection (Fig. 4b). Together, these results show that O-acetylation of the K1 capsule does not influence UPEC bladder colonization in mice.

### O-acetylation increases K1 UPEC survival against human neutrophils

A hallmark of the mouse UTI model is the rapid influx of neutrophils into the bladder post-infection (37). Neutrophils employ multiple antibacterial mechanisms required for the resolution of UTI, including phagocytosis, the release of antimicrobial peptides, generation of reactive oxygen species, and formation of neutrophil extracellular traps (38–40). Previous studies have shown that the K1 capsule is essential for UPEC protection against neutrophil-mediated killing and contributes to the capacity of UPEC to form intracellular bacterial communities (IBCs) within the bladder uroepithelium (41). Thus, to assess the effect of O-acetylation on neutrophil killing in a controlled experimental system that avoids the complexity of the mouse UTI model, we tested the survival of the isogenic strains MS7163, MS7163∆*neuO*, and MS7163*neuO*^CC^ following incubation with human neutrophils. Here, we observed the O-acetylated strains MS7163 and MS7163*neuO^CC^* exhibited significantly increased survival compared to the non-O-acetylated strain MS7163*∆neuO* (Fig. 4c). To further evaluate the contribution of *neuO*-mediated O-acetylation against innate immune killing, we performed a serum bactericidal assay. Here, we observed deletion of the *neuO* gene (MS7163∆*neuO*) led to increased survival in human serum compared to O-acetylated wild-type and complemented strains (Fig. 4d). In these experiments, the bactericidal activity of human serum was confirmed by complete (100%) killing of the K-12 MG1655 strain (Fig. S1). Overall, these results demonstrate that O-acetylation of the K1 capsule confers protection against human neutrophil-mediated killing but contrastingly leads to increased susceptibility to human serum. This is consistent with the role of phase variation in promoting different phenotypes.

## DISCUSSION

O-acetylation of the K1 polysialic acid capsule in UPEC is controlled by the CUS-3 phage-encoded phase-variable NeuO O-acetyltransferase. By curating a large genomic data set of K1 *E. coli*, we assessed the prevalence, phase status, and function of the *neuO* gene. We show the *neuO* gene is a common feature in the ST95 clone and exists in phase-ON and phase-OFF states, reflecting active phase switching. We further show NeuO-mediated O-acetylation confers diverse phenotypes, ranging from protection against phage and neutrophil killing to increased susceptibility against human serum, revealing its multifaceted role in UPEC survival.

By curating a large collection of K1 *E. coli* genomes (*n* = 8,659), we showed the *neuO* gene is present in 43.5% of K1 *E. coli*, a finding consistent with a previous study that analyzed a smaller data set (19). The high prevalence of *neuO* (and thus the CUS-3 phage) in ST95 aligns with its conserved K1 genotype (3, 28), which is estimated to have emerged ~250 years ago (3). Intriguingly, this is in contrast to ST1193, which transitioned from a K5 to K1 capsule type ~30 years ago (42), and contained only a small proportion of K1 genomes carrying *neuO* (10.6%). This suggests that the pattern of *neuO* prevalence is associated with CUS-3 phage infection and clonal expansion of successful K1 lineages such as ST95.

Expression of the K1 capsule leads to increased susceptibility to infection and lysis by K1-specific phage (43). K1 phage recognize the Neu5Ac monomers that are the building blocks of the K1 capsule (44). Here, we showed O-acetylation of the K1 capsule reduces susceptibility to a lytic K1 phage, consistent with a previous study that used a different strain-phage combination (16). It appears that NeuO-mediated O-acetylation of the K1 capsule is a mechanism of phage superinfection exclusion, whereby the CUS-3 phage encodes the *neuO* gene to O-acetylate the K1 capsule, preventing recognition and infection by similar K1-specific bacteriophage. We speculate that O-acetylation of the K1 capsule may also generate novel receptor motifs that newly evolved phage could preferentially recognize. Thus, NeuO-mediated O-acetylation could impact phage-driven ecology in multiple ways, and it remains to be determined how this phenotype may more broadly impact horizontal gene transfer in K1 *E. coli*.

The impact of NeuO-mediated O-acetylation on virulence is complicated by its phase-variable expression. We addressed this problem by generating a set of isogenic strains, including a *neuO* mutant (MS7163∆*neuO*) and a locked *neuO* phase-ON strain (MS7163*neuO*^CC^). While mutation of *neuO* did not alter colonization of the mouse bladder, we showed that expression of NeuO does enhance survival against neutrophil killing. Although neutrophil influx is characteristic of infection of the bladder in experimental mice, ST95 K1 UPEC can resist this innate immune response (41), and thus a more nuanced experimental design was used to investigate the impact of O-acetylation. This involved a direct killing assay employing *ex vivo* human neutrophils, where we observed a protective effect mediated by K1 O-acetylation. Given the complexity of UTI, including the establishment of IBCs that shield bacteria from innate immune attack (41), this is not surprising and is in line with previous work that showed NeuO-mediated O-acetylation modulates immune evasion of macrophages (11).

The interpretation of these results has some limitations, for example, the design of the mouse UTI experiments and known differences between mouse and human neutrophils (45). First, our experimental design employed mixed 50:50 infections to assess relative survival; however, we note this may mask the contribution of O-acetylation toward neutrophil-mediated immunity. Indeed, since half of the inoculum expresses an O-acetylated capsule, we cannot rule out the possibility that this may be sufficient to alter the immune environment of the bladder in a way that limits our ability to observe differences. Second, O-acetylation of the K1 capsule has been shown to reduce recognition by Siglecs 5/7/11/14 on human macrophages (11), receptors that are not conserved in mice (46). These species-specific differences in Siglec receptors may also contribute to the observed differences between our human neutrophil killing assays and mouse UTI experiments.

We further explored the role of K1 capsule O-acetylation and immune system interaction through a serum killing assay. Our results show that O-acetylation of the K1 capsule increased susceptibility to human serum, despite its protective effect against neutrophil killing. These findings align with a previous study that demonstrated K1 *E. coli* recovered from the bloodstream of neonatal rats expressed non-O-acetylated polysialic acid, although that study did not directly assess the impact of O-acetylation (17). In contrast, Yang et al. reported enhanced virulence of a serially passaged phase-ON *neuO* variant compared to a primarily phase-OFF isogenic *neuO* parent strain in a neonatal mouse infection model (11). Differences in UPEC strains, *neuO* phase status, and infection models may account for discrepancies between these studies. An independent challenge UTI study comparing the spatial and temporal patterns of colonization of K1 UPEC *neuO* phase-ON and phase-OFF strains may provide deeper insight into the contribution of K1 capsule O-acetylation in pathogenesis and dissemination to the bloodstream.

In conclusion, our findings indicate that phase variation of *neuO* enables UPEC to adapt to different niches. The ability to reversibly switch between O-acetylated and non-O-acetylated K1 capsule states is likely to enhance UPEC survival across diverse host and environmental settings.

## SUPPLEMENTAL MATERIAL

Data S1 jb.00610-25-s0001.xlsx Assembled K1 capsule data with Kaptive typing and neuO BLAST analysis.

Tables S1 and S2; Figure S1 jb.00610-25-s0002.pdf Table S1. Strains used in this study. Table S2. Primers used in this study. Figure S1. Human serum killing assay for K-12 strain MG1655 demonstrating killing activity.

ASM does not own the copyrights to Supplemental Material that may be linked to, or accessed through, an article. The authors have granted ASM a non-exclusive, world-wide license to publish the Supplemental Material files. Please contact the corresponding author directly for reuse.
