# Supplementary material for: NeuO-mediated O-acetylation of uropathogenic Escherichia coli K1 capsule enhances resistance to phage and neutrophil killing
Source: J Bacteriol. 2026 Feb 10;208(3):e00610-25. doi: 10.1128/jb.00610-25 (PMC13001214; doi:10.1128/jb.00610-25)

Supplementary Materials

Supplementary Table 1. Strains used in this study.

Strain ID	Details	Reference
**MS7163**	Wild-type MS7163, O-acetylated capsule	(26)
**MS7163Δ*neuO***	Non-O-acetylated capsule	This study
**MS7163*neuO*^{CC}**	O-acetylated capsule	This study
**MS7163*kpsD*::Cm**	Capsule deficient control	This study

Supplementary Table 2. Primers used in this study. Lower case letters represent nucleotides homologous to the Cm cassette of the pKD3 vector. The 5’ 6-Carboxyfluorescein (6-FAM) fluorophore is represented by /56-FAM/ in the table.

Primer ID	Sequence (5’ to 3’)	Description
**11572**	ATAAATGGCTGAACGCTGTC	Forward primer; *neuO* screening
**11573**	TATTTCTCACGGACTCATGG	Forward primer; *neuO* amplifying
**11574**	ggaataggaactaaggagga TCTTAACATATTAGTATCCTACGT	Reverse primer; *neuO* amplifying
**11575**	cctacacaatcgctcaagac TGGGCAAGAACGGATAAAG	Forward primer; *neuO amplifying*
**11576**	TGCTGACGCTATTGAAGTG	Reverse primer; *neuO amplifying*
**11577**	TTGTGATCAAACAAATCGGC	Reverse primer; *neuO screening*
**11594**	ATGCTTCGCTTAAAGACGC	Forward primer; *neuO cassette*
**11595**	ggaataggaactaaggagga TTGTAAAACGACGGCCAGTG	Reverse primer; *neuO cassette*
**11596**	CGCGAATCTTGCGTCTTTAA TCTTAACATATTAGTATCCTACGT	Forward primer; *neuO cassette stitching*
**3746**	tcctccttagttcctattcc	Forward primer; Cm cassette amplifying
**3747**	gtcttgagcgattgtgtagg	Reverse primer; Cm cassette amplifying
**12008**	/56-FAM/CACATAAAGAAGTAAGTACATTGG	Primer for fragmentation analysis to amplify repeat region
**11527**	GCTATTTACAAGATCTCCAC	Primer for fragmentation analysis to amplify repeat region

Supplementary Figure 1. Human serum killing assay for K-12 strain MG1655 demonstrating killing activity. Bar graph shows CFU/ml of bacteria prior to incubation with human serum (time 0) and at 1 h post-incubation (n = 4 biological replicates).

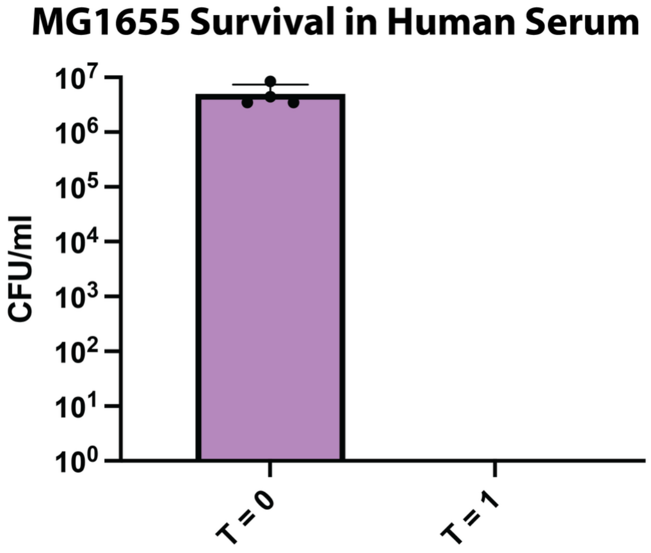

Supplement: Tables S1 and S2; Figure S1 — Table S1. Strains used in this study. Table S2. Primers used in this study. Figure S1. Human serum killing assay for K-12 strain MG1655 demonstrating killing activity. [file jb.00610-25-s0002.pdf]
